# Does the trained immune system play an important role in the extreme longevity that is seen in the Sardinian blue zone?

**DOI:** 10.3389/fragi.2022.1069415

**Published:** 2022-12-19

**Authors:** Mark J. Soloski, Michel Poulain, Giovanni M. Pes

**Affiliations:** ^1^ Division of Rheumatology, Department of Medicine, Johns Hopkins School of Medicine, Baltimore, MD, United States; ^2^ IACCHOS Université Catholique de Louvain, Estonian Institute for Population Studies, Tallinn University, Tallinn, Estonia; ^3^ Dipartimento di Medicina, Chirurgia e Farmacia, University of Sassari, Sassari, Italy

**Keywords:** trained immunity, Sardinia, Italy, longevity blue zone, infection, lifestyle

## Abstract

Villages in the island of Sardinia in the Mediterranean that display exceptional longevity are clustered within a defined mountainous region. Because of their unique location we hypothesize that these villages had a unique infectious disease exposure relevant to the observed successful longevity. These highland villages had a significant exposure to malaria in the first half of the 20th century after which malaria was eliminated due to vector control mechanisms. In addition, there is likely a high incidence of *Helicobacter pylori* infections among shepherds in Sardinia, the primary occupation of many living in the LBZ, as well as helminth infections among children. This suggests that individuals living in the LBZ had a unique infectious disease exposure. Specifically, we hypothesize that the continued high exposure of residents in the LBZ to these infectious agents prior to the 1950s lead to the generation of a uniquely trained (or imprinted) immune system. Once some of these diseases were eliminated in the latter half of the century, individuals within the LBZ were equipped with a trained immune system that was uniquely capable of not only responding effectively to common infections but also responding in a manner that maximized maintaining tissue health. In addition, there are lifestyle factors that also favor such a trained immune system. This hypothesis may help explain the slow progression of chronic immune mediated diseases as well as other chronic non-transmissible age-related diseases seen in the Sardinian LBZ and serve as a template for future studies that support or refute this hypothesis.

## Introduction

1. An Overview of the Longevity Blue Zone (LBZ) in Sardinia.

The Sardinian LBZ is in the central-eastern mountainous region of Sardinia where there is a cluster of villages in which there is a carefully documented high frequency of individuals exhibiting exceptional longevity (Poulain et al., 2004). Researchers developed a measurement termed the Extreme Longevity Index (ELI) to help assess the level of longevity among different villages (Poulain et al., 2004). The ELI is equivalent to the probability of any person born in each municipality to reach 100 years of age. To date there are six villages, where the age of the oldest-old has been fully validated by in depth analysis and documentation (termed the deep or restricted blue zone). There are an additional eight villages that have been analyzed but not yet fully validated (the extended Blue Zone) (Poulain et al., 2020). All fourteen villages are in the Ogliastra and Barbagia subregions (the Sardinian LBZ), display a high ELI, and have been extensively studied over the last 20 years.

The exceptional longevity that is seen in the Sardinian LBZ is thought to be a relatively new phenomenon, observed in the 20th century. This is supported by a review of evidence from the time of Roman occupation that found no evidence for exceptional longevity at that time (Floris et al., 2021).

The comparative analysis of villages in Sardinia with high and low ELI have revealed several important non-genetic factors that are likely contributors to longevity within the Sardinian LBZ. The data collected include assessments of nutritional patterns, psychosocial measurements of wellbeing, stress, physical/motor activity levels, occupation as well as geospatial data (Poulain et al., 2011; Salaris et al., 2012; Pes et al., 2013; Pes et al., 2015; Fastame et al., 2018; Pes et al., 2018; Pes et al., 2020; Pes et al., 2021b; Fastame et al., 2021; Poulain et al., 2021; Fastame, 2022). Collectively, these data revealed that involvement in pastoralism (largely shepherding), high terrain inclination, high physical activity, low body mass index (BMI), successful stress coping mechanisms and high sense of wellbeing were consistently associated with successful aging within the Sardinian LBZ. Nutritional patterns were also examined in several studies and the overall conclusion is that the non-western diet high in grains, fruit and vegetables, moderate consumption of goat and sheep (not cow) based products and some animal derived proteins was associated with longevity (Nieddu et al., 2020).

A particularly interesting and novel feature that has been revealed in studies on individuals in the Sardinian LBZ is the high frequency of male representation with a male/female ratio close to 1 (Poulain et al., 2011; Pes et al., 2013; Pes et al., 2015). This stands in contrast to other studies on longevity where females overwhelming dominate and indicates that the Sardinian LBZ is unique. Studies examining the factors influencing male longevity in the LBZ concluded that factors that impact energy expenditure factors such as occupational activity (pastoralism) and levels of physical activity contribute to male longevity.

There has been considerable interest in the genetic analysis of individuals in Sardinia as the island population exhibits a high frequency of many monogenic, polygenic and autoimmune diseases (Sardu et al., 2012; Lettre and Hirschhorn, 2015). An examination of SNPs and whole genome sequencing of individuals in Sardinia has found that individuals within the Ogliastra region have a unique genetic makeup (Piras et al., 2012; Chiang et al., 2018). However, the identification of a unique genetic footprint that is linked to longevity has proven elusive and recent studies have argued that the genetic contribution to longevity *per se* may be much smaller than previously thought (Dato et al., 2017; Kaplanis et al., 2018; Ruby et al., 2018).

2. Infectious Agents as an Environmental Factor in the Sardinian LBZ.

Environmental factors play an important part in human health and likely contribute to longevity. Environmental Factors can include climate, exposure to man-made and natural substances and exposure to infectious agents. Currently information on the exposure to natural or man-made substances within the Sardinian LBZ is not available and awaits further study. However, infectious diseases have clearly made an impact on the health and wellbeing of the population in Sardinia. Exposure to infectious agents have been linked to long term chronic illnesses that impact longevity. We suggest that the infectious disease exposure history of residents in the Sardinian LBZ needs to be considered as a contributing factor to the high frequency of extreme longevity seen there. Below we will describe several types of infectious disease and share, when available, demographic information on how these diseases may have impacted the Sardinian LBZ.

*Helicobacter pylori* is a Gram-negative bacterium that can colonize the gastric compartment. Infection can cause gastric illness and can be linked to the development of gastric cancer, increased risk for cardiovascular disease but is most often asymptomatic (Malfertheiner et al., 2018; Zhang et al., 2020; Dore et al., 2022). This pathogen is largely transmitted by the oral ingestion of contaminated food particles and has been shown to have an increased prevalence in shepherds, the primary occupation linked to longevity in the Sardinian LBZ (Dore et al., 1999a; Dore et al., 2001). Also, transmission of *H. pylori* is thought to be transmitted *via* contaminated goat milk (Dore et al., 1999b; Dore et al., 2001). Recently, pastoralism (shepherding) has been used as a proxy of *Helicobacter pylori* infection in the study of gastric cancer in Sardinia and identified a high incidence of gastric disease overlapping with the Sardinian LBZ (Pes et al., 2021a). In addition, in a recent systematic review of the occupational risk of acquiring *H. pylori* infection, it was found that those active in agriculture are among several groups to have a higher risk of infection (Kheyre et al., 2018) Therefore, it is reasonable to suggest that *H. pylori* infection was and perhaps still is a common feature among residents of the Sardinian LBZ.

Soil based helminth infections were very common in Sardinia (Palmas et al., 2003). Prior to the 1950s, because Sardinians existed on a subsistence economy, some have suggested that its profile was comparable to many developing nations (Attanasio et al., 1985). The most widespread infection was the intestinal helminth *Enterobius vermicularis* (pin worm) and reinfection was common among children.

In addition to soil-based helminth infections, animal transmitted infections need to be considered. Echinococcosis, caused by the helminth *Echinococcus granulosus* has long been endemic in Sardinia and only recently have steps been taken to lower it incidence (Attanasio et al., 1985; Craig and Larrieu, 2006). Echinococcus is transmitted between dogs and domestic livestock in particular sheep. It is known that human transmission occurs frequently in communities engaged in pastoralism. To our knowledge, detailed geospatial or epidemiological records on the precise incidence of various helminth infections in Sardinia are not available. However, a recent study examining samples from 19th century cesspits using classical techniques coupled and deep sequencing approaches, identified a range of helminths at such sites (Chessa et al., 2020). Collectively, this indicates that exposure to helminths was historically wide-spread in Sardinia, and we would argue that they were particularly high in regions engaged in intensive pastoralism such as the villages in the Sardinian LBZ.

**FIGURE 1:**
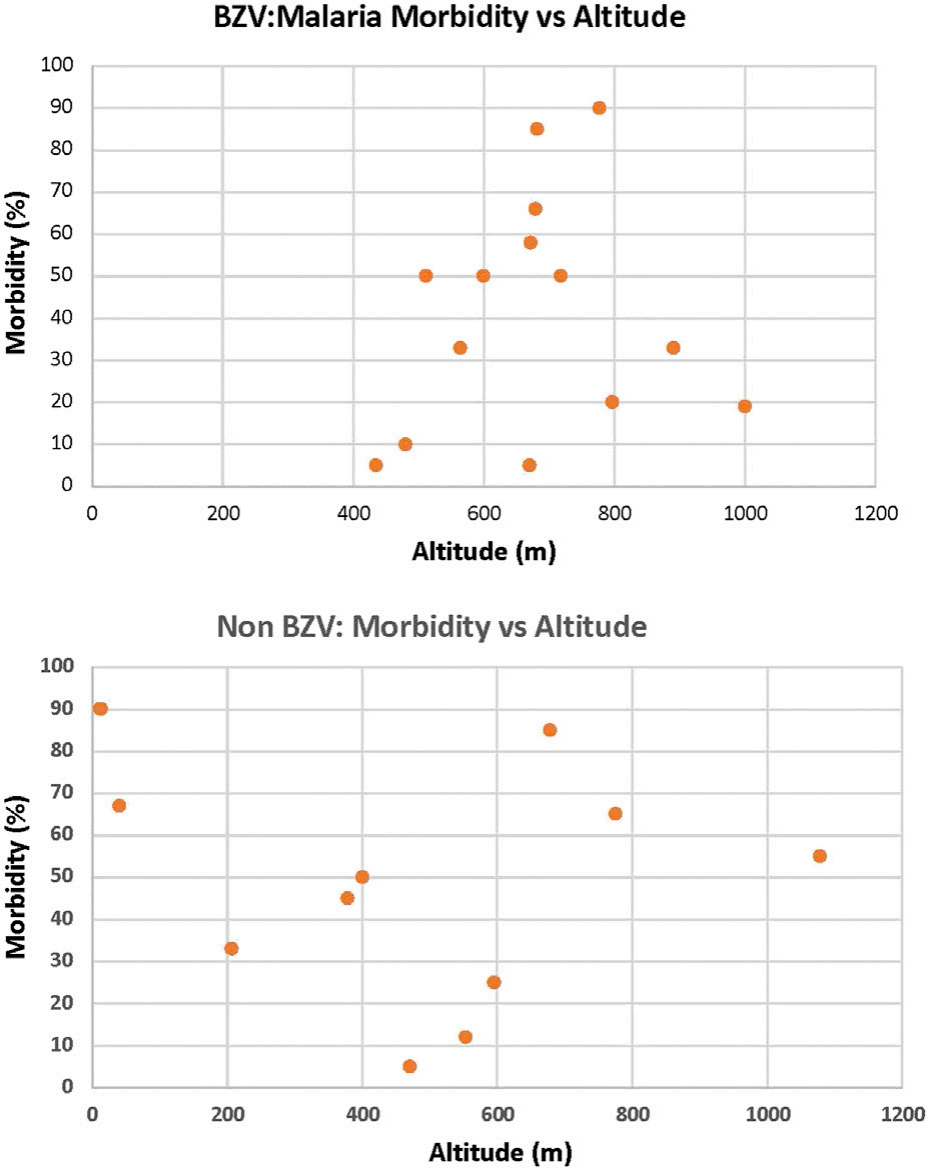
Altitude is not a factor for the Incidence of Malaria in the LBZ. Utilizing the data of Fermi (Fermi, 1934, 1938, 1940), there was no significant correlation with malaria incidence among LBZ villages (top) nor in villages in the same region that do not display longevity (bottom).

It is well known that malaria was, for centuries, a major health problem in Sardinia with a high level of morbidity and mortality (Tognotti, 1996). Detailed studies conducted by Claudio Fermi in the 1930s identified villages with high and low incidence of malaria and found there was regional heterogeneity (Fermi, 1934, 1938, 1940). Interestingly, an analysis comparing altitude and malaria incidence reported that villages residing a >800 m had a low incidence while, low lying and coastal regions had a high incidence of malaria. (Contu et al., 2009). This variation was proposed to be due to the low levels of the *Anopheles* vector at higher elevations. We examined the data of Fermi in the context of the Sardinian LBZ villages and found that, while the altitude of these villages varied from 434–1000 m, there was no significant correlation with malaria incidence among this cluster of villages nor in villages in the same region that do not display longevity (Figure 1). Therefore, at least with the subregion of Ogliastra where these villages reside, the relationship between altitude and malaria morbidity does not hold and we conclude that malaria was widespread with that region. However, it is important to note that, in the second half of the 20th century, due to a major vector eradication program, malaria was essentially eliminated from Sardinia (Tognotti, 2009). This would indicate that individuals in the Sardinian LBZ were exposed to malaria during the first half of the 20th century, but that exposure ended soon thereafter.

Tuberculosis was also a major health problem in Sardinia in the early 20th century. Detailed regional data as to the incidence of TB in Sardinia, similar to the data collected by Fermi, is not available. However, a paper by Sabbatani comparing the incidence of tuberculosis in early 20th century Italy, a chart is presented indicating that the Ogliastra region in Sardinia was a region of high incidence in 1927 (Sabbatani, 2005). Also, analysis of the incidence of mortality due to tuberculosis in the 20th century in Sardinia showed a steady death rate in the 1950s then a clear decline likely due to the advent of successful treatments and changes in public health practices. Therefore, perhaps as we discuss above for malaria, individuals in the Sardinian LBZ were significantly exposed to tuberculosis during the first half of the 20th century, but that exposure ended soon thereafter.

Lastly, we would like to consider disease exposure as it relates to the levels of sanitation. As mentioned above, some have typified hygiene in Sardinia during the late 19th and early 20th century as comparable to many developing nations (Attanasio et al., 1985). This would indicate general sanitation practices in the Sardinian LBZ may have provided the setting for exposure to several enteric infectious diseases. General hygiene practices began to improve in Sardinia during the early 20th century as evidenced by the decline of typhoid fever (Bianchi et al., 1968). However, it is also thought that the Ogliastra and Barbagia subregions lagged in this transition. Interestingly, in the studies of Fermi, he reported that 13/14 of the designated LBZ villages lacked a sewer system (“fognatura”) at that time (Fermi, 1938, 1940). This would suggest, as above, that individuals within the Sardinia LBZ were likely exposed, in the first half of the 20th century, to a range of bacterial and/or viral driven enteric infections.

Collectively the above information indicates that individuals that display extreme longevity within the Sardinian LBZ were likely to have had a unique and novel infectious disease exposure history. We hypothesize that this history has impacted the immune systems of these individuals and is a contributing factor in their ability to age successfully. The immune mechanisms that may underlay this contribution will be discussed in the next section.

## What is trained immunity and why might it be relevant to longevity in the LBZ in Sardinia

The concept of trained immunity is relatively new and is based on the initial observation that exposure of monocytes to *Candida albicans* leads to functional reprogramming and protection against reinfection (Quintin et al., 2012). Subsequently a long list of microbial substances (BCG vaccine, low levels of LPS, *etc.*), signaling *via* pattern recognition receptors, have been shown to train monocytes/macrophages to provide enhanced protection against the same or a different set of pathogens (Netea et al., 2016; Jeljeli et al., 2019; Bekkering et al., 2021). Trained immunity has also been termed “innate immune memory” as the initial exposure targets innate immune cells that will inform the downstream adaptive (B and T-cell based) immune response. This process has been observed in plants, invertebrates and mammals including humans. Trained immunity is likely highly relevant in humans as it has been observed that prior BCG vaccination may initiate innate immune responses that favorably impact the recovery from COVID-19 (Netea et al., 2020).

The mechanisms that drive trained immunity involve epigenetic and metabolic reprogramming of innate immune cells *via* histone modification, DNA methylation, modulation of miRNA and the expression of long-noncoding RNAs (Netea et al., 2016; Jeljeli et al., 2019; Bekkering et al., 2021). Initially this reprogramming was described in macrophages/monocytes, but recent work has shown that other innate immune cells such as, dendritic cells, NK cells, ILC1 and ICL2 cells. Trained immunity in ICL2 cells is particularly notable as these innate cells have been shown to respond to tissue injury signals and mediate tissue repair and remodeling.

The duration of trained immunity is not short-lived as a single microbial exposure has been shown, in animal models and in the human setting, to last month’s up to a year (Netea et al., 2016). Interestingly, hematopoietic progenitor cells (HPCs) have also been shown to undergo reprogramming. This “central trained immunity” is especially relevant as myeloid cells have a short half-life in circulation and that this reprogramming of progenitor cells can produce a steady supply of peripheral trained innate immune cells in the host explaining, in part, the observed duration of trained immunity (Netea et al., 2016).

Recent data have also revealed that several non-immune cell types can produce cytokines and/or anti-microbial factors and can display a trained immunity phenotype (Hamada et al., 2018). To date, these include synovial fibroblasts, endothelial cells, epithelial cells, and vascular smooth muscle cells (Oosting et al., 2016). Collectively these observations indicate that exposure of a host to microbial products can produce trained immunity phenotypes in a wide range of innate immune and non-immune cells, globally impacting the responses of a range of tissues to subsequent immune triggers.

Trained immunity can have a range of outcomes that have been termed by some adaptive *versus* maladaptive programs (Netea et al., 2016). Adaptive programs, on one end of the spectrum are characterized by responses that limit tissue damage, promote tissue regeneration, mucosal tolerance and promote non-specific innate immunity that can limit morbidity and mortality due to infection. On the other end of the spectrum are maladaptive responses which can promote pathogen clearance but can also result in hyper-inflammation in tissues, tissue damage and lead to increased morbidity and mortality. Pro-inflammatory (maladaptive) and anti-inflammatory (adaptive) type trained immunity likely exist side by side but one may dominate over the other in individuals or groups of individuals. Maladaptive responses have been proposed to impact a range of chronic disease conditions such as atherosclerosis, immune mediated fibrosis, intestinal homeostasis, cardiovascular disease, autoimmune diseases, neuroinflammation as well as cancer. Therefore, trained immunity can have beneficial as well as detrimental effects on overall human health. The factors that impact where on the spectrum an individual’s trained immunity lies are complex. They include the route and dose of microbial exposure, whether the microbial exposure is one time verse repeated, genetic factors, as well as lifestyle modifiers such as nutrition lifestyle, stress, and physical activity levels to name a few. It is likely that the balance of beneficial *versus* detrimental effects of trained immunity will vary individually and perhaps among demographically distinct groups. Nevertheless, this balance is highly likely to have a significant impact on human health.

We propose that trained immunity is a factor that is promoting longevity in the designated LBZ in Sardinia. It is important to note that trained immunity has been proposed previously and discussed as an important factor contributing to extreme longevity (Franceschi et al., 2017; Santoro et al., 2021). This insight is based on the study of a demographically diverse group of individuals who have successfully aged 90–100 + years. The study of these individuals has shown that they have avoided, delayed, or have slow progression of many age related chronic diseases such as cardiovascular disease, diabetes, the onset of dementia and cancer (Franceschi et al., 2018). Since many of these diseases are driven by immune factors it has been put forth that long lived individuals have a trained immune system that is skewed toward an anti-inflammatory (adaptive) slowing and/or preventing the age limiting effects of immune mediated tissue damage. The immunological features of this population have been extensively characterized and there are several unique features of innate immune cells in successfully aged individuals that are supportive of a role for trained immunity (Santoro et al., 2021).

In the case of the Sardinian LBZ, this may represent a unique opportunity to argue for, investigate and test the role of trained immunity in extreme longevity. The LBZ represents a cohort of individuals in a small geographically defined area who, as discussed above, have a unique pathogen exposure history as they lived through the 20th and into the 21st century. In addition, this population has been investigated in detail and shown to have several unique nutritional, behavioral and physical attributes. We argue that collectively all these features will favor the development of adaptive trained immunity and disfavor the development of a maladaptive trained immunity.

Inhabitants of the Sardinian LBZ were likely exposed to a wide range of helminth infections due to their geospatial location and pastoral occupation. Helminth infections are well known for not inducing a proinflammatory immune process but instead induce strong type-2 immune reactions and driving the induction of M2 macrophages (Ludwig-Portugall and Layland, 2012; Murray, 2017). This ability to modify the host environment promotes the survival of host and parasite. The Hygiene Hypothesis argues that the novel immune reactions triggered by helminths may, in part, be responsible for the overall lower incidence of severe autoimmune diseases in regions with high helminth exposure and has even suggested new therapeutic avenues for immune mediated diseases (Palmas et al., 2003; Coakley et al., 2016; Maizels, 2016). Interestingly, recent work has also shown that antigens from helminths can induce metabolic reprograming of macrophages, suppressing a proinflammatory response (Zakeri et al., 2022).

Infection with the Gram-negative bacterium *H. pylori*, as argued above, is also likely a common infection in residents in the Sardinian LBZ. Many Gram-negative bacteria can stimulate a strong proinflammatory response. Notably, *H. pylori* stands in contrast, inducing only a relatively weak response thus promoting the establishment of a persistent infection. Studies have revealed this is due to several unique immune evasion mechanisms driven by bacterial virulence genes (Neuper et al., 2022; Sijmons et al., 2022). These mechanisms include survival in a hostile environment, the altering (reprogramming of?) macrophage and dendritic cell function, promotion of the release of the anti-inflammatory cytokine IL-10 and the promotion of T regulatory cell development (Kaebisch et al., 2014; Kabisch et al., 2016; Altobelli et al., 2019; Frauenlob et al., 2022). For example, while exposure of human monocytes to live *H. pylori* can initially drive the expression of a mix of pro- and anti-inflammatory cytokines, the longer-term innate memory response favors production of anti-inflammatory cytokines (Frauenlob et al., 2022). The net result is that *H. pylori* colonization of the gut results in the down regulation of a proinflammatory response and the longer-term promotion of an anti-inflammatory environment that ensures pathogen survival.

Prior to 1950, Sardinia had the highest incidence of malaria in the Mediterranean basin. Malaria was caused by both *Plasmodium falciparum* and *Plasmodium vivax* strains and produced, for centuries, a high degree of morbidity and mortality (Tognotti, 2009). The high levels of malaria on Sardinia were considered to be a significant barrier to the human and economic development of the island. Infection with Plasmodium species induces a strong pro-inflammatory response, especially in the blood stage (Crompton et al., 2014; Silva et al., 2020). This inflammatory response has been suggested by some to be of the maladaptive type in that it can promote severe tissue injury (Cunnington et al., 2013; Penha-Goncalves, 2019). Furthermore, recent studies have demonstrated that Plasmodium can reprogram monocytes, and this is accompanied by metabolic and epigenetic changes as well as the production of proinflammatory cytokines. (Schrum et al., 2018; Walk et al., 2020). Collectively this information clearly indicates that exposure to Plasmodium (often repeatedly) drives a maladaptive type of trained immunity accompanied by tissue injury. However, this stimulus would have ended after the 1950s. As mentioned above, the precise duration of trained immunity is not known but it is also not considered to be of the same duration as adaptive immune memory (decades or more). Therefore, we would predict that the malaria driven maladaptive trained immunity would wane over time. This may allow either for slow turnover of programmed innate immune cells or for their “reprogramming” to display more adaptive features.

The encounter of the human host to *Mycobacteria tuberculosis* has been shown to activate a range of immune cell types, including macrophages and other innate immune cells (Kaufmann, 2002; O'Garra et al., 2013; de Martino et al., 2019). This encounter drives a potent pro-inflammatory response that can result in significant tissue mediated injury (Kaufmann, 2002; O'Garra et al., 2013; de Martino et al., 2019). Currently direct evidence for the induction of trained immunity by the encounter of virulent Mtb with innate immune cells is not available. However, it has been well documented that monocytes exposed to the attenuated Mtb strain *Bacillus* Calmette-Guérin (BCG) do undergo functional and epigenetic reprogramming characteristic of trained immunity (Steigler et al., 2019; Ferluga et al., 2020; Kong et al., 2021; Zhou et al., 2021). Therefore, we consider it likely that, during tuberculosis, trained innate immune cells with a proinflammatory/maladaptive phenotype emerge. Like malaria above, as Mtb exposure significantly declined after the 1950s, this maladaptive trained immunity would wane.

In addition to infectious disease exposure, there is an increasing awareness of the influence of non-infectious/lifestyle modifiers of trained immunity (Furman et al., 2019; Bajpai and Nahrendorf, 2021). For example, stress induced noradrenaline may induce proinflammatory biased functional and epigenetic changes in myeloid cells (van der Heijden et al., 2020). Using an atherosclerosis prone mouse model, it was determined that providing a prolonged western style diet induced systemic inflammation and the functional and epigenetic reprogramming of myeloid cells (Christ et al., 2018). In support of this, monocytes from individuals with symptomatic atherosclerosis displayed a proinflammatory trained immunity phenotype (Bekkering et al., 2016). In a recent study it was shown that changes in diet, sleep, exercise and stress levels altered the epigenetic age of individuals (Fitzgerald et al., 2021). Lastly, it has also been shown that exercise can influence trained immunity by promoting the development of an anti-inflammatory trained immunity (Zhang et al., 2021). It is important to mention these because a prominent feature of individuals experiencing longevity within the Sardinian LBZ is that they display several shared dietary, behavioral and physical activity features that would disfavor the development of proinflammatory maladaptive trained immunity and favor the generation of anti-inflammatory adaptive trained immunity.

Interestingly, a recent study examining the changes in T-cell subset frequency that accompanies aging found that terminally differentiated T-cell increased with age and that this change was accelerated by psychosocial stress and is a contributor to immune aging and poor health (Klopack et al., 2022). It seems that a lifestyle that minimizes such stressors may reduce immune aging and improve long term health.

In sum, residents within the Sardinian LBZ not only had a unique infectious disease exposure during the first half of the 20th century but they also had and continue to exhibit distinctive life-style modifiers. Collectively, we argue, these factors contribute to longevity by promoting a trained innate immune system that, when engaged by current environmental triggers, will initiate a host response that is largely non-proinflammatory in nature favoring low levels of tissue injury.

## Concluding remarks and future questions

We propose that those within the Sardinian LBZ that exhibit enhanced longevity display have enhanced adaptive trained immunity features that promotes this longevity. This is due, at least in part, to the infectious disease history and the continuing lifestyle features of this community. Furthermore, we are not proposing that there is an absence of pro-inflammatory trained immunity but that the levels of this type of response is modulated. In support of this is the observations, as has been observed in other studies on extreme longevity, that in the Sardinian LBZ there is either avoidance of onset or slow progression of many age-related immune mediated chronic diseases (Santoro et al., 2021).

**FIGURE 2:**
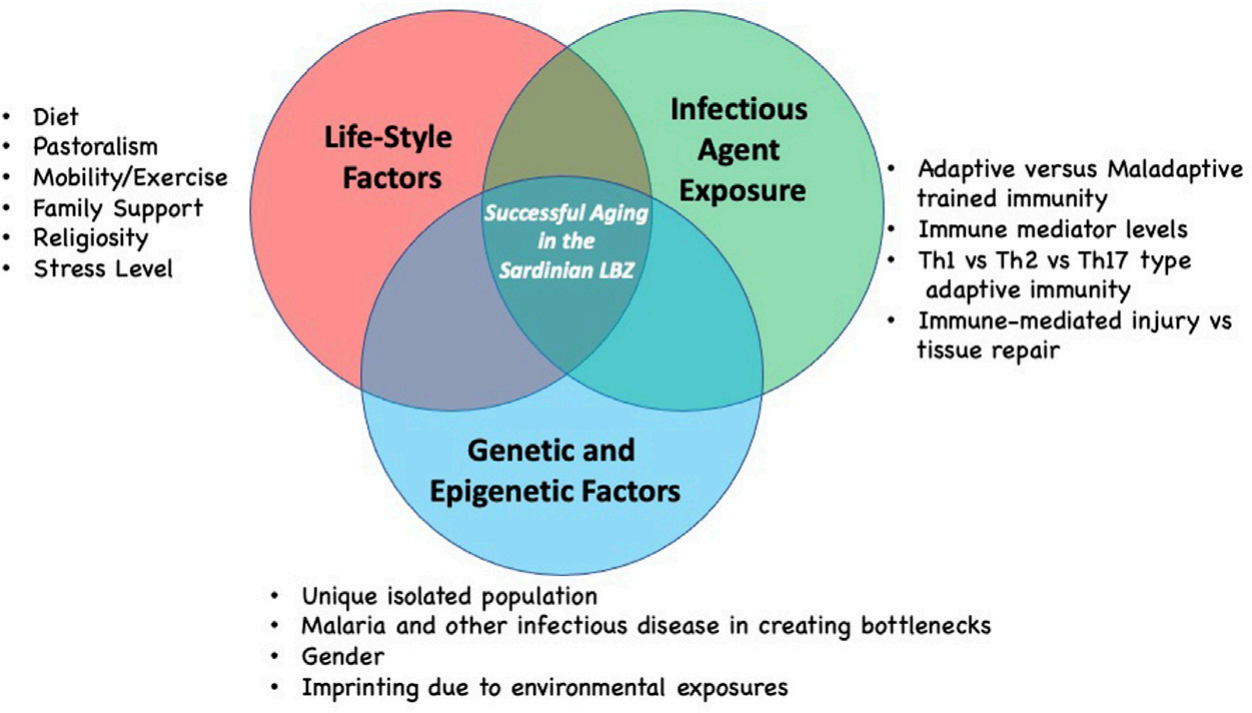
Hypotheses regarding the complexity of factors that may contribute to longevity as seen in the Sardinian LBZ. Each factor group may contribute in differing ways to successful aging based on lived experience of individuals. Future contributors to successful longevity in the Sardinian LBZ may still be identified.

There are likely several other factors that contribute to longevity in the Sardinian LBZ in addition to the infectious disease history and life-style, including the role of the family environment, support systems as well as genetics (Figure 2). Regarding the latter, the genetic analysis of individuals who display extreme longevity has not revealed to date a clear genotype associated with successful aging. At best, it is likely that successful aging is a complex genetic trait and the genetic contribution to longevity may vary considerably from cohort to cohort. As mentioned above the genetic analysis of Sardinians has revealed a distinct genetic fingerprint in individuals in the region of Ogliastra. However, it is not clear that this is linked to longevity. We would suggest that a genetic analysis, modeled after the recent study examining sociodemographic and other variables in nonagenarians with aged-matched non-agenarians from two regions with differing overall longevity levels, could identify such a fingerprint (Pes et al., 2020). Once identified these could be tested on other longevity cohorts.

Based on the arguments put forward above, we would make several predictions regarding the immune features of individuals successfully aging within the LBZ.

1) Tissue macrophages, and potentially blood monocytes would express features of tissue repair and/or resolving/regulatory macrophages. This would include the expression of soluble factors that promote tissue repair and regeneration such as TGF-α, TGF-β, PDGF, IGF-1, AREG, and IL-10.
2) The levels of mediators produced by proinflammatory macrophages such as TNF-α, IL-1β and IL-6 would be lower. We are aware that previous studies have yielded different conclusions about the roles of different functional subsets of macrophages in successful aging (Hearps et al., 2012; Costantini et al., 2018). However, the cohorts used were geographically distinct (Italy *versus* Australia) and contained few individuals over 90. We suggest that the study of successfully aging individuals in the Sardinian LBZ represents a unique opportunity to address this issue.
3) Successfully aging individuals would display high levels of tissue resident and/or circulating ILC2 cells. These innate immune cells have been strongly associated with tissue repair and regeneration and secrete the cytokines IL-4, IL-5, IL-9, and IL-13.
4) Promoter and/or enhancer regions of the genes mentioned above will have epigenetic markers reflective of their expression status. For example, repressive markers (i.e., H3K4me3) would be found on proinflammatory genes while enhanced gene expression marks (i.e., H3K27ac) would be found on genes promoting tissue repair and regeneration.
5) Based on the study by Klopack et al., we predict that individuals within the Sardinian LBZ that display longevity will display, as a group, higher levels of naïve T-cell and lower levels of terminally differentiated T-cell *versus* a matched study group (Klopack et al., 2022).
6) We predict that the observation of longevity within the Sardinian LBZ is a transient feature of this unique and geographically distinct people. In other words, as the current longevity group begins to pass away, longevity frequency will decrease to a level seen in the regions of Sardinia outside the LBZ. This has been proposed previously (Poulain et al., 2020). We would argue that for those born after 1950 the landscape has and continues to change. These individuals within the Sardinian LBZ have experienced vaccinations, a different infectious disease exposure (most recently COVID-19) and saw changes in hygiene and public health practices. In addition, there are clear changes in nutritional, economic and life-style practices that may impact healthy extreme longevity.

Funding Sources: MJS was supported by grant 076855631 from the Fulbright U.S. Scholar Program/Fondazione Con Il Sud. GMP and MP did not receive any specific grant from funding agencies in the public, commercial or not-for-profit sectors for this work.

## Data Availability

The data analyzed in this study is subject to the following licenses/restrictions: There are no restrictions. Requests to access these datasets should be directed to Mark J. Soloski, mski@jhmi.edu.
